# Association between physical activity bout length and physical functioning in older adults using absolute and relative intensity thresholds: a longitudinal cohort study

**DOI:** 10.1093/gerona/glag219

**Published:** 2026-09-03

**Authors:** Matti Hyvärinen, Antti Löppönen, Timo Aittokoski, Soren Brage, Tomas Gonzales, Taina Rantanen, Laura Karavirta

**Affiliations:** Gerontology Research Center, Faculty of Sport and Health Sciences, University of Jyväskylä, Jyväskylä, Finland; Gerontology Research Center, Faculty of Sport and Health Sciences, University of Jyväskylä, Jyväskylä, Finland; Gerontology Research Center, Faculty of Sport and Health Sciences, University of Jyväskylä, Jyväskylä, Finland; MRC Epidemiology Unit, University of Cambridge, Cambridge, United Kingdom; MRC Epidemiology Unit, University of Cambridge, Cambridge, United Kingdom; Gerontology Research Center, Faculty of Sport and Health Sciences, University of Jyväskylä, Jyväskylä, Finland; Gerontology Research Center, Faculty of Sport and Health Sciences, University of Jyväskylä, Jyväskylä, Finland; (Medical Sciences Section)

**Keywords:** Exercise intensity, Accelerometer, Physical fitness, Functional capacity, Physical activity energy expenditure

## Abstract

**Background:**

Defining moderate-to-vigorous physical activity (MVPA) relative to individual capacity, instead of absolute intensity cut-points, may provide a more meaningful representation of physical activity in relation to age-related decline in physical functioning. We examined whether associations between MVPA energy expenditure (MVPAEE) and changes in physical functioning differ when MVPA is defined using absolute versus relative intensity cut-points, and whether bout length modifies these associations in older adults.

**Methods:**

This 4-year follow-up study included 295 community-dwelling participants aged 75, 80, and 85 at baseline. Physical functioning was assessed using 6-min walking distance (6MWD) and five-times sit-to-stand test (FTSTS) time. MVPAEE was measured using thigh-worn accelerometers combined with chest-worn electrocardiography. MVPAEE accumulated in bouts of varying lengths was defined using both absolute intensity cut-points and cut-points relative to individual capacity. Associations between baseline MVPAEE and changes in physical functioning were examined using linear regression models and compositional data analysis.

**Results:**

Physical functioning declined during the follow-up. Higher relative MVPAEE was associated with a smaller decline in 6MWD (β = 0.27, 95% CI [0.08, 0.46]). Compositional data analyses showed that a higher proportion of MVPA relative to light-intensity activity energy expenditure was associated with a smaller decline in 6MWD (β_ilr_ = 0.25-0.31), independent of bout length. Absolute MVPAEE and bout-specific accumulation were not associated with changes in 6MWD or FTSTS.

**Conclusions:**

The total volume and intensity of physical activity relative to an individual’s capacity, rather than bout length, may be key factors in counteracting short-term declines in physical functioning in older adults.

## Introduction

Physical functioning, defined as the ability to perform the physical tasks of everyday living, is a key determinant of health and independence in older adults.^1^ The well-known age-related decline in physical functioning, partly driven by reductions in aerobic capacity and muscular strength and power, has been associated with increased risk of falls,^2^ sarcopenia and frailty,^3^ cardiovascular disease,^4^ and mortality.^4^^,^^5^ Therefore, understanding the factors that support the maintenance of functional capacity across the adult lifespan is essential for promoting health and preserving independence in aging populations.

Aerobic physical activity (PA), which is characterized by continued work of skeletal muscles and a transient increase in energy expenditure,^6^ may contribute to maintaining physical functioning in old age.^7^ According to the specificity principle of physical activity, adaptations to PA are specific to the activity mode.^8^ Thus, the current guideline for older people to engage in 150 min of at least moderate intensity activity,^9^ may help maintain aerobic capacity, ie, the ability to sustain an activity bout. The prolonged duration of an activity bout has traditionally been an essential component of aerobic exercise prescription.^10^ Yet, research has not provided clear evidence to support any minimum threshold for the bout duration, and thus, the previous requirement of 10 min for an activity bout has been removed from the PA guidelines.^6^^,^^9^

Unlike the PA assessment methodology based on self-report, the state-of-the-art wearable technology enables the detection of PA accumulated in shorter bouts,^11^ which comprises most of the everyday activity of adults.^12^ Especially in older adults, PA is often accumulated in shorter bouts, potentially due to increased fatigability and reduced physical functioning.^13^ Yet, it remains unclear whether physical functioning, including both aerobic endurance and muscular function, is associated with PA bout length. While most epidemiological studies have not found longitudinal evidence of any additional benefit from accumulating PA in longer versus shorter bouts beyond total activity volume,^14^ some recent findings suggest that PA bout length may play a role in determining health outcomes.^15^

In addition to overcoming some limitations of self-reported methods, accelerometers and other wearable sensors have changed the way PA intensity is assessed. Unlike questionnaires, wearable sensors can estimate activity intensity based on movement-derived measures of energy expenditure and also provide detailed information on PA accumulation patterns, including PA bout length. Questionnaires, such as the Global Physical Activity Questionnaire, define intensity as perceived effort, described as “increases in breathing or heart rate,”^16^ which, at any absolute intensity level, are determined by individual aerobic capacity. On the other hand, accelerometers are calibrated to assess the rate of energy expenditure, often as metabolic equivalents of task (MET), which is a measure of absolute intensity. The prevailing use of accelerometry to assess absolute intensity, rather than relative to individual capacity, has been accepted as a limitation and a necessary tradeoff to enable large-scale data collections using wearable sensors, where individualized intensity assessment may not be feasible.^17^ However, it remains unclear how the shift from relative to absolute intensity assessment affects the observed associations between aerobic PA and physical function in older age. In older age, aerobic capacity is decreased, which contributes to both physical function and daily PA.^10^ To date, the association between bout duration and PA using individualized intensity cutoffs remains unexplored.

Activities performed closer to an individual’s maximal capacity result in a shorter time to exhaustion.^18^ Thus, individuals with higher aerobic capacity can maintain any absolute intensity for a longer duration than individuals with lower maximum capacity. The relationship between intensity and time to exhaustion is curvilinear, so that the additional physical effort per unit of duration increases exponentially with intensity. Intensity and duration of an activity bout are thus closely intertwined, especially in the moderate-to-vigorous domain. In older adults, even the intensity of walking may approach their maximal capacity.^19^ To account for the increased physiological demands associated with longer activity bouts, intensity needs to be individually assessed. Therefore, the aim of this study was to investigate associations between age-related decline in physical functioning, assessed through aerobic capacity and lower-limb strength and power, and moderate-to-vigorous physical activity (MVPA) bout length under free-living conditions in older adults. Since the internal load of an activity bout is determined by its duration and intensity relative to an individual’s aerobic capacity rather than absolute intensity,^20^ we hypothesized that activity bout length would be independently associated with changes in physical functioning only when activity intensity is quantified in relative terms.

## Methods

We used longitudinal data from a population-based observational AGNES study (Active aging--resilience and external support as modifiers of the disablement outcome). The study included 3 age cohorts: 75, 80, and 85 years old, and the study protocol has been previously published.^21^ The Research Ethics Committee of the Central Finland Health Care District (currently named Wellbeing Services County of Central Finland) provided an approval statement on the AGNES baseline protocol on the 23rd of August 2017, and follow-up on the 8th of September 2021 (Reg. no 14U/2017 and 4U/2021). Participants were required to provide written informed consent. The baseline and follow-up measurements were conducted in 2017-2018 and 2021-2022, respectively.

The detailed flowchart of the baseline recruitment process has been presented previously.^22^ In brief, 2 791 individuals were drawn from the population register and contacted, of whom 37% of them consented to participate. From this sample of 1 021 participants (57% women), 910 individuals agreed to participate in the laboratory assessments and were thus also invited to take part in device-based PA monitoring in free-living conditions.^23^ To those who agreed to wear a thigh-mounted accelerometer, we also offered an electrocardiogram (ECG) recorder unless the participant had an active implantable medical device, such as a cardiac pacemaker (*n* = 19). We excluded participants with less than 3 days of wearable data and eventually obtained combined accelerometry and heart rate (HR) data from 409 participants. Of these, 295 (62% women) took part in the follow-up measurements, comprising the final analytical sample for this study (Figure S1).

### Outcomes

Aerobic capacity was assessed by walking distance (6MWD) derived from a modified 6-min walk test conducted at baseline and follow-up. The walking test was performed in an indoor 20-m corridor at a self-selected usual pace.^24^ Lower-limb strength and power were assessed using the completion time of the five-times-sit-to-stand test,^21^ which was administered in the participants’ homes. Relative change from baseline, calculated in relation to the baseline value, and absolute within-person change in 6MWD and five-times sit-to-stand test (FTSTS) time were used as outcome variables in the analyses.

### Exposure

PA energy expenditure (PAEE) of usual daily living was assessed only at baseline, and the details of the method have been described previously.^23^ Briefly, a thigh-worn accelerometer and a chest-worn ECG recorder were used to record raw acceleration and ECG signals, respectively, for 3 to 7 days in free-living conditions. Raw triaxial acceleration was collected using UKK RM42 accelerometers (UKK Terveyspalvelut Oy, Tampere, Finland) at 100 Hz with acceleration range of ±16 g. The data were calibrated to local gravity,^25^ pre-processed to mean amplitude deviation (MAD) in non-overlapping 5-s epochs,^26^ and resampled to 10-s epochs. The data were visually checked day-by-day to ensure that only days with complete 24-h data without non-wear time were included in the subsequent analysis. ECG was recorded using eMotion Faros 180 (Bittium Corporation, Oulu, Finland), and the recordings were analyzed with commercially available and medically certified Awario arrhythmia analysis algorithms (Awario, Heart2Save, Kuopio, Finland).^27^

PAEE was estimated separately for each 10-s epoch from accelerometry-based data using MAD values and HR data using an individual calibration equation based on a self-paced walking test.^23^^,^^28^^,^^29^ PAEE based on accelerometry- and HR-based estimates were then combined using branched equation modeling to obtain the final PAEE estimates.^30^ In essence, the model gives greater weight to accelerometry-based PAEE estimation when acceleration and HR are low (due to the known fluctuation in HR at low levels regardless of PA), and a larger weight to HR when it is above the flex point and accompanied by physical movement, implemented as described elsewhere.^31^ In addition, whenever HR was not available due to noise or transient arrhythmia in the ECG signal, only PAEE was estimated using the accelerometry-based estimate.

Moderate-to-vigorous PA energy expenditure (MVPAEE) was assessed using both absolute and relative intensity cut-points. The absolute moderate-intensity cut-point corresponded to 3 METs (ie, 10.5 mL/kg/min),^32^ equivalent to a PAEE of 142 J/kg/min, representing 2 METs over the basal metabolic rate.^33^ The estimated PAEE at a self-selected usual walking pace during the 6-min walk test was used as the basis for deriving an individual relative MVPAEE cut-point.^24^^,^^29^ To ensure comparability between the absolute and relative definitions of MVPAEE, the walk-test-derived PAEE values were scaled so that their mean corresponded to the absolute moderate-intensity cut-point. Specifically, each walk-test-derived PAEE value was multiplied by the ratio of the absolute moderate-intensity cut-point to the mean walk-test-derived PAEE (187 J/kg/min). The resulting scaled values were used as the individual relative MVPAEE cut-points. All activity with an intensity below the MVPA cut-points was considered light physical activity.

A bout of MVPA was defined as a period of activity during which at least 80% of the total duration was accumulated above the moderate-intensity cut-point.^34^ As a generalization of the previously used method, we did not set a minimum duration for a bout. Thus, the minimum bout duration was equal to the epoch length of 10 s. Finally, we derived variables representing total MVPAEE accumulated in bouts of varying minimum lengths (30 s and 1, 5, 10, and 30 min).

### Covariates

Sex and date of birth were available in the sample specifics drawn from the Digital and Population Services Agency (https://dvv.fi/en). Body height was measured with a stadiometer, and body mass was assessed using an electronic scale integrated into the InBody720 (Biospace, Seoul, Korea) device. Body mass index (BMI) was computed as body mass divided by squared body height.

### Missing data

There were 355 missing values across all variables and time points, corresponding to 4% of all data points used in the statistical analyses. The proportion of missing values across variables in each time point ranged from 0% to 20%. The highest proportions of missing data were observed in the follow-up 6-min walk test and body weight and height measurements, whereas missingness in other variables was minimal. Missing data resulted from invalid or absent measurements, as well as unclear or incomplete questionnaire responses. Based on the mechanisms, the missing data were assumed to be missing at random (MAR), and multiple imputation was used to address missingness.

Multiple imputation was conducted in R^35^ (R Foundation for Statistical Computing, Vienna, Austria) using the standard settings of the “mice” package^36^ with 50 iterations conducted for each of the 50 imputed datasets. The full dataset used for imputation included age, sex, body mass, body height, BMI, 6MWD and FTSTS at both timepoints, change scores in 6MWD and FTSTS during the follow-up, and all PAEE variables. For the imputation of each target variable, the predictor set comprised all non-derived variables measured at the same timepoint together with the corresponding measurement of the target variable from the other timepoint. Passive imputation was applied to derived variables (BMI and change scores in 6MWD and FTSTS). The parameters of substantive interest were estimated separately in each imputed dataset and then combined using Rubin’s rules.^37^ The “pool” function from the “mice” package was used to combine results. The pooled results were compared with complete case analyses, and the findings did not differ meaningfully between the methods.

### Statistical analysis

Associations between total MVPAEE and changes in physical functioning during follow-up were examined using linear regression models adjusted for age, sex, BMI, follow-up time, and total PAEE. Analyses were conducted separately for absolute and relative MVPA cut-points, with 6MWD and FTSTS time analyzed as separate outcomes.

To investigate the associations of MVPAEE accumulated in different bout lengths and physical functioning, the isometric log-ratio (ilr) approach for compositional data analysis with a total (tCoDA) was applied.^38^ In tCoDA analyses, total PAEE was partitioned into 3 components: PAEE from bouted MVPA, non-bouted MVPA, and light physical activity. The tCoDA approach was chosen because it enabled examination of whether the distribution of total PAEE across these activity components was associated with changes in physical functioning, while accounting for differences in overall physical activity level.^38^

The ilr transformation was implemented in R using packages “compositions”^39^ and “zCompositions”^40^ with a sequential binary partition basis^41^ using the following the equations:


$$V_{1}=\ln\left(\frac{C_{1}}{C_{2}}\right)$$

$$V_{2}=\ln\left(\frac{C_{3}}{C_{1}C_{2}}\right)$$

where $C_{1}$, $C_{2}$, and $C_{3}$ correspond to bouted PAEE, non-bouted PAEE, and light physical activity PAEE, respectively.

Finally, the ilr-transformed components were included in the regression model together with the natural logarithm of total PAEE. This resulted in the following regression equation, presented here without covariates for simplicity:


$$Y_{i}=\beta_{0}+\beta_{1}V_{1i}+\beta_{2}V_{2i}+\beta_{3\;}\ln(T_{i})+\varepsilon_{i}$$

where $V_{1}$ and $V_{2}\;$are the ilr-transformed components, and T is total PAEE. To facilitate interpretation of the results, the signs of the β-coefficients for the second ilr coordinate ($V_{2}$) are reversed in “the Results section” (ie, their additive inverses were reported) so that positive coefficients correspond to a higher proportion of total MVPAEE relative to the light physical activity PAEE.

Separate models were created for absolute and relative MVPA cut-points and for 6MWD and FTSTS time, with changes during the follow-up in the latter used as the outcome variables. The confounders included in the models were age, sex, BMI, and the follow-up time. Model assumptions were tested using residual plots, Q-Q plots, and correlation analyses to assess the model assumptions. All analyses were conducted in R.^35^

## Results

### Characteristics of the study population

The average age of the participants at baseline was 77.9 years, and the duration of the follow-up was 3.9 years (Table 1). Participants were slightly overweight, with a mean BMI of 27.5 kg/m^2^ at baseline and 26.9 kg/m^2^ at follow-up. With a mean body mass of 74.4 kg, the average total PAEE in the study population was 2 321 kJ per day, with a standard deviation (*SD*) of 707 kJ. Moderate-to-vigorous activities accounted for 30% of this total, regardless of the used moderate-intensity cut-point. On average, physical functioning declined during the follow-up period, with a mean decrease of 51 meters (*SD* 46) in the 6MWD and an increase of 1.0 s (*SD* 4.3) in STS time. Relative to baseline values, the changes corresponded to −11.3% (*SD* 10.8) and +12.4% (*SD* 35.1) for 6MWD and STS time, respectively.

**Table 1:** Characteristics of the study population.

	Baseline		Follow-up		Change^a^	
	*n*	*M* (*SD*)	*n*	*M* (*SD*)	*n*	*M* (*SD*)
**Age (years)**	295	77.9 (3.1)	295	81.8 (3.1)	295	3.9 (0.3)
**Body mass (kg)**	292	74.4 (12.5)	252	73.1 (12.3)	249	−2.5 (3.7)
**Body mass index (kg/m^2^)**	292	27.5 (4.2)	252	26.9 (4.0)	249	−0.7 (1.4)
**Six-minute walking distance (m)**	291	432 (75)	236	393 (73)	236	−51 (46)
**Five times sit-to-stand test time (s)**	291	12.1 (3.6)	295	13.3 (5.1)	291	1.0 (4.3)
**Sex^b^**	295					
Men		38 (111)				
Women		62 (184)				
**Total PAEE (kJ/kg/day)**	295	31.2 (9.5)				
**Absolute moderate-to-vigorous physical activity cut-points**						
MVPAEE (kJ/kg/day)	295	9.4 (7.0)				
MVPAEE in bouts over 30 s (kJ/kg/day)	295	8.1 (6.8)				
MVPAEE in bouts over 1 min (kJ/kg/day)	295	7.5 (6.6)				
MVPAEE in bouts over 5 min (kJ/kg/day)	295	5.9 (6.1)				
MVPAEE in bouts over 10 min (kJ/kg/day)	295	5.1 (5.9)				
MVPAEE in bouts over 30 min (kJ/kg/day)	295	3.4 (5.2)				
**Relative moderate-to-vigorous physical activity cut-points**						
MVPAEE (kJ/kg/day)	291	9.4 (6.6)				
MVPAEE in bouts over 30 s (kJ/kg/day)	291	8.0 (6.3)				
MVPAEE in bouts over 1 min (kJ/kg/day)	291	7.3 (6.1)				
MVPAEE in bouts over 5 min (kJ/kg/day)	291	5.7 (5.7)				
MVPAEE in bouts over 10 min (kJ/kg/day)	291	4.8 (5.4)				
MVPAEE in bouts over 30 min (kJ/kg/day)	291	3.2 (4.8)				

Data are *M* (*SD*) unless otherwise specified.

Abbreviations: MVPAEE, moderate-to-vigorous physical activity energy expenditure; PAEE, physical activity energy expenditure; SPPB, Short Physical Performance Battery.

a Only for participants with both baseline and follow-up measurements.

b Data are % (*n*).

Over 85% of the participants engaged in MVPA in longer bouts than 10 min, regardless of the moderate-intensity cut-point used (Figure 1). However, a slightly greater proportion of participants met the relative intensity cut-point for minimum bout lengths ranging from 30 s to 10 min than the absolute cut-point. In contrast, clearly fewer participants engaged in MVPA in bouts longer than 60 min when using the relative (29%) compared to absolute (61%) cut-points. As shown in Figure S2, the correlation between total absolute and relative MVPAEE was 0.86 (95% CI [0.83, 0.89]). The figure further illustrated that participants with a longer 6-min walking distance accumulated more MVPAEE when absolute moderate-intensity cut-points were applied, whereas relative cut-points resulted in more comparable MVPAEE estimates across different levels of physical functioning.

**Figure 1:**
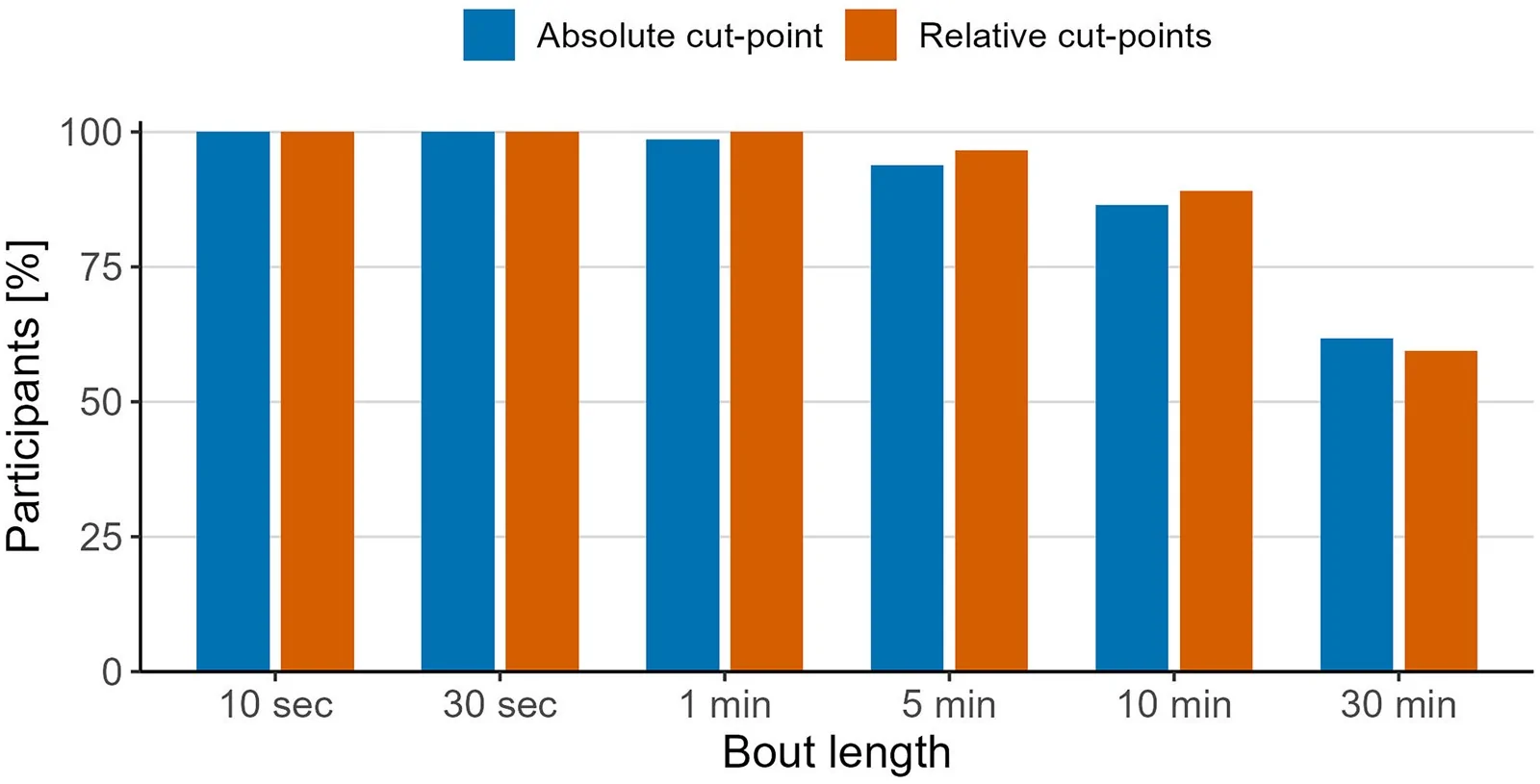
Proportion of participants engaging in moderate-to-vigorous physical activity with different minimum bout lengths.

### Associations between total moderate-to-vigorous physical activity and changes in physical functioning

With relative MVPA cut-points, higher total MVPAEE was positively associated with the change in 6MWD relative to baseline value (β = 0.27, 95% CI [0.08, 0.46]) (Table 2), as well as with absolute change (0.41 [0.23, 0.59]) (Table S1). In contrast, when using the absolute cut-point for MVPA, the MVPAEE was not associated with the change in 6MWD. Furthermore, MVPAEE was not associated with the change in FTSTS time during the follow-up regardless of the MVPA cut-points used.

**Table 2:** Linear regression analyses on the associations between total moderate-to-vigorous physical activity energy expenditure and relative changes in measures of physical functioning over the 4-year follow-up (*n* = 295).

	Absolute cut-points		Relative cut-points	
	β	95% CI	β	95% CI
**Relative change in 6-min walking distance**				
MVPAEE	−0.06	[−0.31, 0.19]	0.27**	[0.08, 0.46]
**Relative change in 5 times sit-to-stand test time**				
MVPAEE	0.10	[−0.15, 0.35]	0.15	[−0.03, 0.33]

Models are adjusted for age, sex, body mass index, follow-up time, and total physical activity energy expenditure. Multiple imputation was applied in the analyses.

Abbreviations: β, standardized regression coefficient; MVPAEE, moderate-to-vigorous physical activity energy expenditure.

** *p* ≤ .01.

### Associations of bout-specific moderate-to-vigorous physical activity and changes in physical functioning

The tCoDA models with relative change in 6MWD as the outcome indicated that the proportion of MVPAEE accumulated in bouts was not associated with the change in aerobic capacity during the follow-up, regardless of the minimum bout length or the MVPA cut-points used (Table 3). However, reallocating energy expenditure from light physical activity to MVPA was associated with a smaller decline in 6MWD when using the relative MVPA cut-points and adjusting for the accumulated MVPAEE in bouts, with β_ilr_ varying from 0.25 to 0.31. This reallocation was not statistically significant for absolute MVPAEE with respect to the change in 6MWD. The results were largely consistent when absolute change in 6MWD was used as the outcome (Table S2). For example, using a 10-min minimum bout length, reallocating 2 kJ/kg/day of PAEE from light physical activity to MVPA relative to the sample compositional center corresponded to an estimated 6-meter smaller decline in 6MWD during follow-up when relative MVPA cut-points were used, whereas the corresponding reallocation using absolute MVPA cut-points corresponded to an estimated 3-meter greater decline in 6MWD (Figure S3). Reallocation of PAEE from non-bouted to bouted MVPA, or from light physical activity to MVPA, was not associated with changes in FTSTS time during the follow-up (Table 4, Table S3). However, when using an absolute MVPA cut-point and adjusting the model for accumulation MVPAEE in bouts, the reallocation of PAEE from light physical activity to MVPA tended to be associated with a smaller increase in FTSTS time during the follow-up.

**Table 3:** Compositional linear regression analyses on the associations between physical activity energy expenditure and relative changes in 6-min walking distance over the follow-up period (*n* = 295).

	Absolute cut-points		Relative cut-points	
	β_ilr_	95% CI	β_ilr_	95% CI
**MVPAEE accumulated in bouts over 30 s**				
Bouted vs non-bouted MVPAEE	−0.02	[−0.22, 0.17]	0.03	[−0.15, 0.21]
Total MVPAEE vs light PAEE	−0.11	[−0.47, 0.24]	0.31**	[0.08, 0.54]
**MVPAEE accumulated in bouts over 1 min**				
Bouted vs non-bouted MVPAEE	−0.08	[−0.28, 0.11]	−0.02	[−0.19, 0.16]
Total MVPAEE vs light PAEE	−0.11	[−0.44, 0.23]	0.30*	[0.06, 0.53]
**MVPAEE accumulated in bouts over 5 min**				
Bouted vs non-bouted MVPAEE	0.06	[−0.13, −0.26]	−0.12	[−0.27, 0.03]
Total MVPAEE vs light PAEE	−0.11	[−0.44, 0.22]	0.25*	[0.02, 0.49]
**MVPAEE accumulated in bouts over 10 min**				
Bouted vs non-bouted MVPAEE	0.06	[−0.13, 0.26]	−0.14	[−0.28, 0.00]
Total MVPAEE vs light PAEE	−0.11	[−0.44, 0.22]	0.26*	[0.03, 0.49]
**MVPAEE accumulated in bouts over 30 min**				
Bouted vs non-bouted MVPAEE	0.09	[−0.10, 0.28]	−0.13	[−0.27, 0.01]
Total MVPAEE vs light PAEE	−0.15	[−0.47, 0.17]	0.26*	[0.04, 0.49]

Models are adjusted for age, sex, body mass index, follow-up time, and the logarithm of total physical activity energy expenditure. Multiple imputation was applied in the analyses.

Abbreviations: β_ilr_, regression coefficient from an isometric log-ratio transformed composition; MVPAEE, moderate-to-vigorous physical activity energy expenditure.

* *p* ≤ .05;

** *p* ≤ .01.

**Table 4:** Compositional linear regression analyses on the associations between physical activity energy expenditure and relative changes in five times sit-to-stand test time over the follow-up period (*n* = 295).

	Absolute cut-points		Relative cut-points	
	β_ilr_	95% CI	β_ilr_	95% CI
**MVPAEE accumulated in bouts over 30 s**				
Bouted vs non-bouted MVPAEE	−0.11	[−0.28, 0.06]	−0.10	[−0.28, 0.08]
Total MVPAEE vs light PAEE	−0.24	[−0.51, 0.03]	0.11	[−0.11, 0.33]
**MVPAEE accumulated in bouts over 1 min**				
Bouted vs non-bouted MVPAEE	−0.00	[−0.17, 0.17]	−0.11	[−0.30, 0.08]
Total MVPAEE vs light PAEE	−0.18	[−0.43, 0.08]	0.10	[−0.14, 0.33]
**MVPAEE accumulated in bouts over 5 min**				
Bouted vs non-bouted MVPAEE	−0.02	[−0.18, 0.14]	−0.09	[−0.26, 0.09]
Total MVPAEE vs light PAEE	−0.20	[−0.45, 0.06]	0.10	[−0.35, 0.16]
**MVPAEE accumulated in bouts over 10 min**				
Bouted vs non-bouted MVPAEE	0.09	[−0.06, 0.24]	−0.04	[−0.23, 0.15]
Total MVPAEE vs light PAEE	−0.25	[−0.51, 0.00]	0.07	[−0.23, 0.36]
**MVPAEE accumulated in bouts over 30 min**				
Bouted vs non-bouted MVPAEE	0.16	[0.01, 0.30]	0.01	[−0.17, 0.19]
Total MVPAEE vs light PAEE	−0.28*	[−0.52, −0.03]	0.02	[−0.27, 0.30]

Models are adjusted for age, sex, body mass index, follow-up time, and the logarithm of total physical activity energy expenditure. Multiple imputation was applied in the analyses.

Abbreviations: β_ilr_, regression coefficient from an isometric log-ratio transformed composition; MVPAEE, moderate-to-vigorous physical activity energy expenditure.

* *p* ≤ .05.

## Discussion

In this longitudinal study of older adults, we observed that participants with higher MVPAEE, based on the relative MVPA intensity cut-points, had a smaller decline in aerobic capacity during the 4-year follow-up. However, the accumulation of MVPAEE in bouts was not associated with the changes in functional capacity, regardless of whether MVPA was defined using relative or absolute intensity cut-points.

The current results partly support our hypothesis on the divergent longitudinal associations of relative versus absolute MVPA with physical functioning. The observed smaller decline in 6MWD among participants with higher MVPA levels using relative, but not absolute, cut-points is an important novel finding. We have previously demonstrated in the same population-based sample a positive association between PA minutes above self-selected usual walking intensity and maintaining better walking performance.^42^ However, the intensity of self-selected walking in our sample was, on average, higher than the absolute 3-MET cut-point.^24^ Therefore, the differences in results between absolute and relative PA may have been caused by systematically higher relative intensity thresholds. Thus, the present analysis, in which the average intensity of the relative and absolute cut-points were matched, further indicates that relative rather than absolute assessment is the key to unveiling potential health benefits of PA. Another key advancement compared with our previous study is that MVPA volume was assessed in terms of energy expenditure rather than minutes of MVPA. MVPAEE considers the activity volume within an intensity domain rather than simple classification, as MVPA minutes could be accumulated just above the cut-point up to maximum intensity. Yet, both measures assess aerobic rather than muscular activity, which may explain why the association was significant only for the changes in aerobic capacity (ie, 6MWD) and not for lower-limb strength and power (FTSTS time), according to the specificity principle. The present result is also consistent with the overload principle of PA, which proposes that exercise intensity needs to be sufficient relative to the individual’s level of aerobic capacity to provide significant adaptations.^43^ Thus, even high absolute intensity may be too low in relative terms to benefit individuals with a high baseline level of physical function. On the other hand, the higher relative MVPA, inducing a larger internal load, may translate to a smaller decrease in physical functioning in the longitudinal setting.

We also observed that, contrary to our hypothesis, bout length was not a significant contributor to the change in physical function over the 4-year follow-up, regardless of whether relative or absolute intensity cut-point was used. To our knowledge, this is the first study to investigate the association between the bout length of relative MVPA and a health outcome in a longitudinal design. Most previous studies using absolute MVPA metrics, such as a 6-year follow-up study of Japanese adults over 65 years,^44^ have reported similar longitudinal associations between MVPA and physical function, regardless of MVPA bout length. The advisory committee of PA guidelines has summarized that MVPA bouts of any length contribute to health outcomes.^6^ As a result, the minimum bout duration of 10 min has been removed from the MVPA guideline.^9^ More recently, Schwendinger et al.^45^ demonstrated lower cardiovascular disease mortality in association with longer activity bouts when adjusted for intensity, but not independent of total volume of activity. A recent study examining steps, however, observed that longer usual stepping bouts are independently associated with lower all-cause mortality and CVD incidence.^15^ The independent role of bout duration is difficult to investigate since the amount of bouted and non-bouted activity are highly correlated, indicating that the same people who accumulate larger volumes of activity also perform more activity in longer bouts. We aimed to eliminate the effect of this correlation by using the CoDA approach and adjusting the models for total PAEE, since participants who accumulate more MVPA tend to be more active overall. This adjustment may explain the weaker association between the proportions of MVPA accumulated in bouts and physical functioning when using absolute rather than relative cut-points, as the direction of the association between total MVPA and physical functioning is different for absolute and relative cut-points.

Previous studies have shown that the epoch length and the definition of an activity bout used in the analysis of accelerometry-based data affect the derived PA estimates as well as their relationships with health outcomes.^46^ However, there is no consensus on how an activity bout should be defined, as most movement includes breaks due to traffic, obstacles, distractions, or social reasons. In this study, we used relatively short epochs of 10 s and applied a previously suggested definition for a bout, 80% of the total duration of the bout required above the cut-point.^34^ A break in activity that is proportionate to the duration of the whole bout, in comparison to a fixed or no gap allowed, is grounded in physiology. A short break within a longer activity bout leads to a less significant recovery than within a shorter bout. Another strength is the longitudinal study design, which allowed us to investigate the change in physical functioning over time. In addition, we have previously shown that complementing accelerometry with heart rate may capture activity intensity in a broader range compared to an accelerometry-only method.^47^

There are also limitations that need to be considered. The method for estimating the relative moderate intensity cut-point was individualized based on the 6-min walking test intensity and did not use the more conventional 40% of oxygen consumption reserve or heart rate reserve method.^48^ Yet, for older adults, 6MWD provides an estimation of aerobic capacity,^49^ and it accounts for a large portion of individual variation in PA.^50^ We also matched the average relative cut-point to the generally used MVPA cut-point of 3 METs, and thus, the relative cut-point can be considered a tailored version of the commonly used cut-point, grounded in the definition of moderate intensity in relation to the individual fitness level. Furthermore, the 4-year follow-up period may not have been long enough to capture meaningful changes in physical functioning, and the 1-week physical activity assessment may not have been sensitive enough to detect significant associations, as physical activity levels can vary from week to week and year to year. This study concentrated only on MVPA bouts, while activity bouts may also play a role in other intensity domains. We did not investigate light-intensity activity bouts because heart rate, as a measure of activity intensity, is not suitable for estimating PA intensity at low intensities. In addition, the ilr coefficients derived from the tCoDA approach are not directly interpretable in clinically meaningful units, which may reduce the intuitive interpretation of effect sizes. Finally, although participants were drawn from the population register, those who consented to participate may have been healthier and had better functional capacity than the general population. This potential selection bias may limit the generalizability of the findings to the broader population of older adults.

## Conclusions

This study provides preliminary evidence that higher levels of MVPA defined in relative, but not absolute, terms may be associated with better preservation of aerobic capacity, independent of bout length. These results suggest that maintaining sufficient total volume and relative intensity of physical activity may help counteract short-term declines in physical functioning in older adults aged 75 to 90 years, while the accumulation of activity in longer bouts appears less relevant. However, larger studies with longer follow-up periods and additional health outcomes beyond physical functioning are needed to further investigate the role of physical activity accumulation patterns in promoting health among older adults.

## Supplementary Material

glag219_Supplementary_Data

## Data Availability

The datasets used in this study are not publicly available because EU and Finnish data protection legislation, together with the consent provided by the participants, do not permit open sharing of individual-level data. However, metadata from the AGNES study and pseudonymized data are available to external collaborators upon reasonable request, subject to an agreement on data use and the publication of results (https://doi.org/10.17011/jyx/dataset/83811).
