# Supplementary material for: Association between physical activity bout length and physical functioning in older adults using absolute and relative intensity thresholds: a longitudinal cohort study
Source: J Gerontol A Biol Sci Med Sci. 2026 Sep 3;81(10):glag219. doi: 10.1093/gerona/glag219 (PMC13626715; doi:10.1093/gerona/glag219)
Supplement: Supplementary file 1 [file glag219_supplementary_data.docx]

**SUPPLEMENTAL MATERIAL**

**Association between physical activity bout length and physical functioning in older adults using absolute and relative intensity thresholds: A longitudinal cohort study**

**eFigure 1.** Flowchart of the study

**eFigure 2.** Scatterplot of absolute versus relative total MVPAEE by baseline six-minute walking distance. The Pearson correlation coefficient and its 95% confidence interval are presented in the figure. MVPAEE, moderate-to-vigorous physical activity energy expenditure; r, Pearson correlation coefficient; CI, confidence interval.

**eTable 1.** Linear regression analyses on the associations between total moderate-to-vigorous physical activity energy expenditure and absolute changes in measures of physical functioning over the four-year follow-up (n=295).

**eTable 2.** Compositional linear regression analyses on the associations between physical activity energy expenditure and absolute changes in six-minute walking distance over the follow-up period (n=295).

**eFigure 3.** Predicted change in 6MWD associated with reallocating PAEE from light physical activity to MVPA (Panel A) and from non-bouted to bouted MVPA (Panel B) using a 10-minute minimum bout length, relative to the sample compositional center. The sample compositional center for the absolute MVPA cut-points was 25.9, 4.0, and 1.2 kJ/kg/day for light physical activity, non-bouted MVPA, and bouted MVPA, respectively. The corresponding values for the relative MVPA cut-points were 25.3, 4.4, and 1.4 kJ/kg/day, respectively. 6MWD, six-minute walking distance; MVPA, moderate-to-vigorous physical activity; PAEE, physical activity energy expenditure.

**eTable 3.** Compositional linear regression analyses on the associations between physical activity energy expenditure and absolute changes in five times sit-to-stand test time over the follow-up period (n=295).

**eFigure 1.** Flowchart of the study

**eFigure 2.** Scatterplot of absolute versus relative total MVPAEE by baseline six-minute walking distance. The Pearson correlation coefficient and its 95% confidence interval are presented in the figure. MVPAEE, moderate-to-vigorous physical activity energy expenditure; r, Pearson correlation coefficient; CI, confidence interval.

**eTable 1.** Linear regression analyses on the associations between total moderate-to-vigorous physical activity energy expenditure and absolute changes in measures of physical functioning over the four-year follow-up (n=295).

|  | **Absolute cut-points** | | **Relative cut-points** | |
| --- | --- | --- | --- | --- |
|  | **β** | **95% CI** | **β** | **95% CI** |
| **Absolute change in six-minute walking distance** | | | | |
| MVPAEE | -0.07 | [-0.32, 0.18] | 0.41*** | [0.23, 0.59] |
| **Absolute change in five times sit-to-stand test time** | | | | |
| MVPAEE | 0.10 | [-0.14, 0.34] | 0.14 | [-0.04, 0.32] |

Models are adjusted with age, sex, body mass index, follow-up time, and total physical activity energy expenditure. β, standardized regression coefficient; CI, confidence interval; MVPAEE, moderate-to-vigorous physical activity energy expenditure. Multiple imputation was applied in the analyses. * p ≤ 0.05, ** p ≤ 0.01, *** p < 0.001

**eTable 2.** Compositional linear regression analyses on the associations between physical activity energy expenditure and absolute changes in six-minute walking distance over the follow-up period (n=295).

|  | **Absolute cut-points** | | **Relative cut-points** | |
| --- | --- | --- | --- | --- |
|  | **β_ilr_** | **95% CI** | **β_ilr_** | **95% CI** |
| **MVPAEE accumulated in bouts over 30 seconds** | | | | |
| Bouted vs. Non-bouted MVPAEE | -0.02 | [-0.21, 0.18] | -0.00 | [-0.18, 0.17] |
| Total MVPAEE vs. Light PAEE | -0.14 | [-0.45, -0.16] | 0.48*** | [0.27, 0.70] |
| **MVPAEE accumulated in bouts over 1 minute** | | | | |
| Bouted vs. Non-bouted MVPAEE | -0.05 | [-0.24, 0.14] | -0.05 | [-0.22, 0.12] |
| Total MVPAEE vs. Light PAEE | -0.12 | [-0.41, 0.16] | 0.47*** | [0.25, 0.68] |
| **MVPAEE accumulated in bouts over 5 minutes** | | | | |
| Bouted vs. Non-bouted MVPAEE | 0.07 | [-0.11, 0.25] | -0.18* | [-0.32, -0.03] |
| Total MVPAEE vs. Light PAEE | -0.14 | [-0.42, 0.15] | 0.40*** | [0.19, 0.62] |
| **MVPAEE accumulated in bouts over 10 minutes** | | | | |
| Bouted vs. Non-bouted MVPAEE | 0.11 | [-0.06, 0.28] | -0.21** | [-0.34, -0.08] |
| Total MVPAEE vs. Light PAEE | -0.19 | [0.48, -0.09] | 0.40*** | [0.19, 0.62] |
| **MVPAEE accumulated in bouts over 30 minutes** | | | | |
| Bouted vs. Non-bouted MVPAEE | 0.10 | [-0.06, 0.26] | -0.20** | [-0.33, -0.06] |
| Total MVPAEE vs. Light PAEE | -0.18 | [-0.45, 0.09] | 0.40*** | [0.18, 0.61] |

Models are adjusted with age, sex, body mass index, follow-up time, and the logarithm of total physical activity energy expenditure. β_ilr_, regression coefficient from an isometric log-ratio transformed composition; CI, confidence interval; MVPAEE, moderate-to-vigorous physical activity energy expenditure; Multiple imputation was applied in the analyses. * p ≤ 0.05, ** p ≤ 0.01, *** p < 0.001

**eFigure 3.** Predicted change in 6MWD associated with reallocating PAEE from light physical activity to MVPA (Panel A) and from non-bouted to bouted MVPA (Panel B) using a 10-minute minimum bout length, relative to the sample compositional center. The sample compositional center for the absolute MVPA cut-points was 25.9, 4.0, and 1.2 kJ/kg/day for light physical activity, non-bouted MVPA, and bouted MVPA, respectively. The corresponding values for the relative MVPA cut-points were 25.3, 4.4, and 1.4 kJ/kg/day, respectively. 6MWD, six-minute walking distance; MVPA, moderate-to-vigorous physical activity; PAEE, physical activity energy expenditure.

**eTable 3.** Compositional linear regression analyses on the associations between physical activity energy expenditure and absolute changes in five times sit-to-stand test time over the follow-up period (n=295).

|  | **Absolute cut-points** | | **Relative cut-points** | |
| --- | --- | --- | --- | --- |
|  | **β_ilr_** | **95% CI** | **β_ilr_** | **95% CI** |
| **MVPAEE accumulated in bouts over 30 seconds** | | | | |
| Bouted vs. Non-bouted MVPAEE | -0.07 | [-0.24, 0.11] | -0.09 | [-0.27, 0.09] |
| Total MVPAEE vs. Light PAEE | -0.28* | [-0.54, -0.02] | 0.11 | [-0.12, 0.34] |
| **MVPAEE accumulated in bouts over 1 minute** | | | | |
| Bouted vs. Non-bouted MVPAEE | 0.05 | [-0.12, 0.22] | -0.10 | [-0.28, 0.09] |
| Total MVPAEE vs. Light PAEE | -0.22 | [-0.47, 0.03] | 0.10 | [-0.14, 0.34] |
| **MVPAEE accumulated in bouts over 5 minutes** | | | | |
| Bouted vs. Non-bouted MVPAEE | 0.01 | [-0.15, 0.16] | -0.07 | [-0.24, 0.11] |
| Total MVPAEE vs. Light PAEE | -0.24 | [-0.48, 0.00] | 0.09 | [-0.17, 0.35] |
| **MVPAEE accumulated in bouts over 10 minutes** | | | | |
| Bouted vs. Non-bouted MVPAEE | 0.11 | [-0.03, 0.26] | -0.03 | [-0.22, 0.16] |
| Total MVPAEE vs. Light PAEE | -0.29* | [-0.53, -0.04] | 0.06 | [-0.24, 0.36] |
| **MVPAEE accumulated in bouts over 30 minutes** | | | | |
| Bouted vs. Non-bouted MVPAEE | 0.17 | [0.03, 0.30] | 0.02 | [-0.16, 0.19] |
| Total MVPAEE vs. Light PAEE | -0.27* | [-0.51 -0.04] | 0.01 | [-0.26, 0.29] |

Models are adjusted with age, sex, body mass index, follow-up time, and the logarithm of total physical activity energy expenditure. β_ilr_, regression coefficient from an isometric log-ratio transformed composition; CI, confidence interval; MVPAEE, moderate-to-vigorous physical activity energy expenditure; Multiple imputation was applied in the analyses. * p ≤ 0.05, ** p ≤ 0.01, *** p < 0.001
